# Coding Patterns and Implications for Reimbursement in Foot-and-Ankle Surgery

**DOI:** 10.7759/cureus.81955

**Published:** 2025-04-09

**Authors:** Ryan G Rogero, Carson M Rider, Benjamin J Grear, David R Richardson, Garnett A Murphy, Clayton C Bettin

**Affiliations:** 1 Orthopaedic Surgery and Biomedical Engineering, University of Tennessee Health Science Center-Campbell Clinic, Memphis, USA

**Keywords:** current procedural terminology, medical billing, medical coding, practice management, reimbursement, surgeon education

## Abstract

Introduction: Coding is an essential part of a foot-and-ankle surgeon’s responsibility and can quantify the amount of work done by the surgeon and influence compensation. The purpose of this study was to evaluate the coding patterns and variation among foot-and-ankle orthopedic surgeons and to quantify the potential effects of these on reimbursement using real-life patient cases. Our hypothesis was that there would be large variability between the coding of common foot-and-ankle cases between surgeons, with subsequent effects on reimbursement values.

Methods: A survey consisting of 12 patient cases was administered to all foot-and-ankle, fellowship-trained orthopedic surgeons of a large, combined academic-private practice group. The scenarios included pre-operative diagnostic imaging and reports, intra-operative imaging, and post-operative radiographs. Surgeons were asked which Current Procedural Terminology (CPT) codes would be applied and if any modifiers to these codes would be used. Total work-relative value units (RVUs) and the generated reimbursement values were calculated for each case and respondent using the 2024 Centers for Medicare & Medicaid Services conversion factor ($32.74 per RVU), with the primary procedure reimbursed at 100% and additional procedures reimbursed at 50%.

Results: Five surgeons completed the survey. Among case scenarios, wide variability in CPT coding was demonstrated, with only 33.3% (four out of 12) of cases having at least four of the five respondents in agreement on the primary CPT code, whereas only one case had 100% agreement. Similarly, only 41.7% (five out of 12) of cases had at least four of the five respondents in agreement regarding modifier usage, with only one case having 100% agreement. The total RVU and reimbursement difference between the respondents with the highest and lowest listed RVUs was 216.06 and $3,627.92, respectively.

Conclusion: Large variability exists between foot-and-ankle surgeons when coding common procedures, particularly those involving the midfoot. Surgeons should be aware of these differences and the large effect they can have on quantifying work and reimbursement. Increasing competency with coding and billing should continue to be emphasized in all medical specialties.

## Introduction

Coding is an important part of a foot-and-ankle surgeon’s duties, and it has become more complex in the modern healthcare system. It can quantify both the amount of work done by the surgeon as well as influence compensation. There has been a recent increase in emphasis on improving coding practices in orthopedic surgery, resident education, and other medical specialties [1-4].

Current Procedural Terminology (CPT) codes, created and updated by the American Medical Association (AMA), are used to identify specific procedures performed and guide reimbursement [5]. Current Procedural Terminology modifiers are used to report or indicate that a performed service/procedure has been altered by a specific circumstance but has not changed its definition or code [6]. Both aspects of coding may be influenced by individual practice patterns, reimbursement models, and practice knowledge and literacy regarding coding [7]. Recent studies have demonstrated wide variations in CPT coding practices in various surgical procedures [7-10]. Previous studies have also demonstrated substantial variability between resident and surgeon coding [2,9,11]. No study was found that investigates coding patterns and variability in foot-and-ankle cases among practicing surgeons.

The purpose of this study was to evaluate the coding patterns among foot-and-ankle orthopedic surgeons and quantify their effects on reimbursement using real-life patient cases. Our hypothesis was that there would be large variability between the coding of common foot-and-ankle cases between surgeons, with subsequent effects on reimbursement values.

Of note, this study was presented as an electronic poster at the 2024 American Orthopaedic Foot & Ankle Society (AOFAS) meeting held September 11-14 [12] and is scheduled to be presented at the podium of the 2025 Mid-America Orthopaedic Association (MAOA) Annual Meeting on April 12 in San Antonio, Texas.

## Materials and methods

This study was conducted at the University of Tennessee Health Science Center-Campbell Clinic, Memphis, TN. Institutional Review Board approval and informed consent were not required for this study. A survey consisting of 12 commonly encountered real deidentified patient cases was administered to all foot-and-ankle, fellowship-trained orthopedic surgeons of a large, combined academic-private practice orthopedic group. The survey was created by the senior author, who has an extensive background in coding and practice management. The survey included various foot-and-ankle pathologies, including both trauma and elective cases (Table 1). 

**Table 1:** Patient case descriptions NC: naviculocuneiform; ORIF: open reduction and internal fixation; TMT: tarsometatarsal

Case	Description
1	2^nd^ metatarsophalangeal joint dislocation with metatarsal head overload undergoing open reduction and metatarsal shortening (Weil) osteotomy
2	Posterior tibial tendon insufficiency with gastrocnemius contracture and spring ligament tear undergoing minimally invasive medial displacement calcaneal osteotomy, spring ligament repair, Kidner procedure, and Strayer procedure
3	Trimalleolar ankle fracture undergoing hindfoot nailing with medial malleolus resection
4	5^th^ metatarsal base fracture undergoing ORIF
5	Subacute Lisfranc and 2^nd^ TMT joint dislocations undergoing midfoot arthrodesis
6	Maisonneuve injury undergoing syndesmotic fixation and deltoid ligament repair
7	Bimalleolar ankle fracture non-union undergoing hardware removal, revision fixation, and bone grafting
8	Charcot neuroarthropathy midfoot reconstruction
9	2^nd^-5^th^ metatarsal fracture-dislocations and medial/middle NC dislocations undergoing 2^nd^/3^rd^ TMT joint arthrodeses, medial/middle NC arthrodeses, and pinning of 4^th^/5^th^ TMT joints
10	Talar osteochondral lesion with lateral ankle instability undergoing ankle arthroscopic debridement, arthroscopic-assisted microfracture of talar osteochondral lesion with bone marrow aspirate placement, and lateral ankle ligament reconstruction
11	Trimalleolar ankle fracture undergoing fixation of lateral and posterior malleoli
12	Calcaneus osteomyelitis with nondisplaced fracture and overlying abscess undergoing debridement

**Figure 1:**
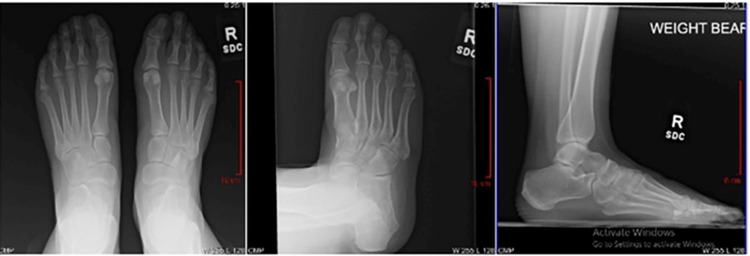
Example of mock patient scenario (Case 1) provided to surgeons; Pre-operative radiographs demonstrating 2nd metatarsophalangeal joint dislocation with metatarsal head overload.

**Figure 2:**
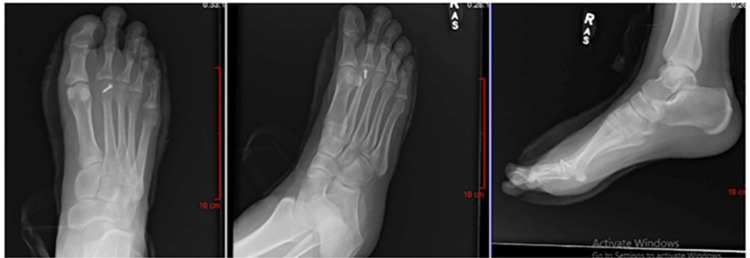
Example of mock patient scenario (Case 1) provided to surgeons; Post-operative radiographs demonstrating open reduction and metatarsal shortening (Weil) osteotomy.

**Figure 3:**
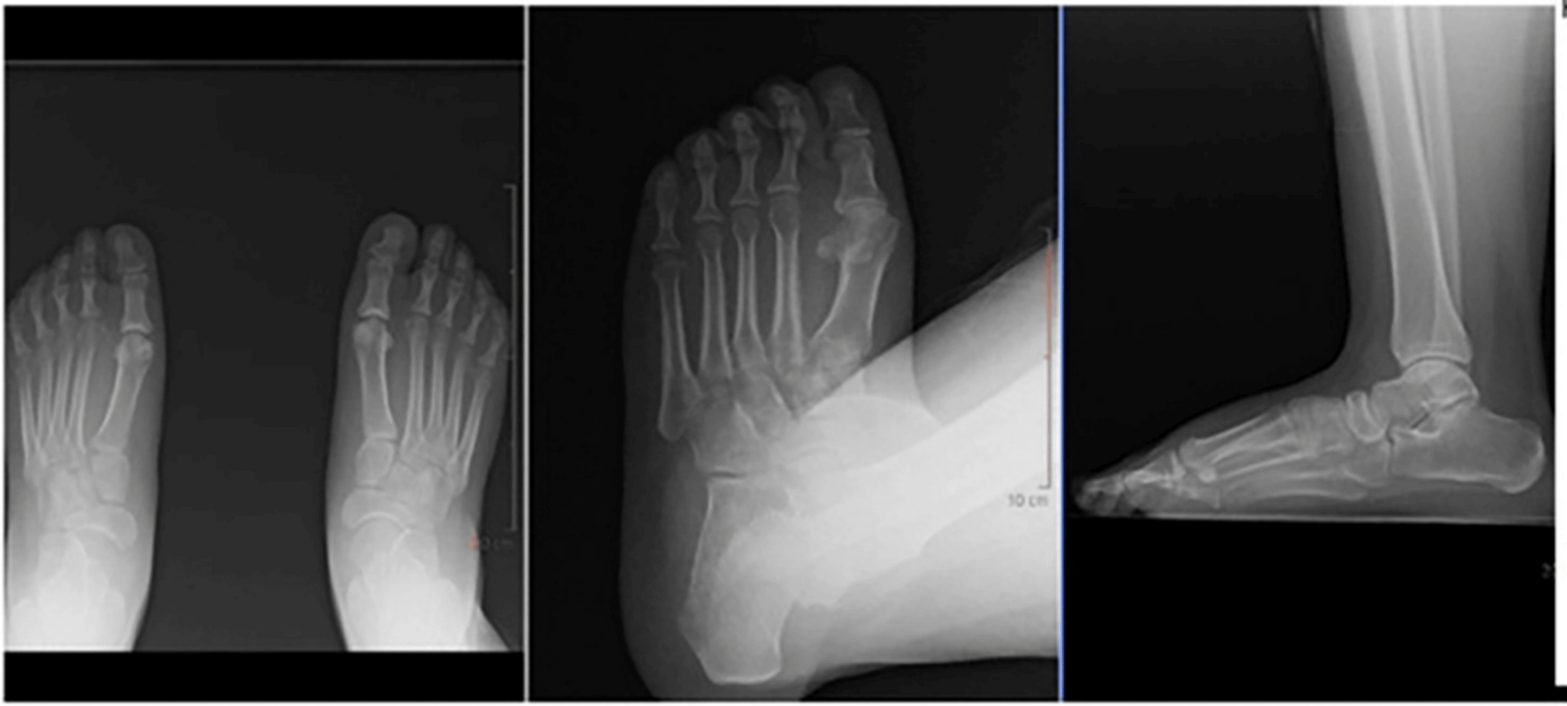
Example of mock patient scenario (Case 5) provided to surgeons; Pre-operative radiographs demonstrating subacute Lisfranc injury and 2nd tarsometatarsal joint dislocation.

**Figure 4:**
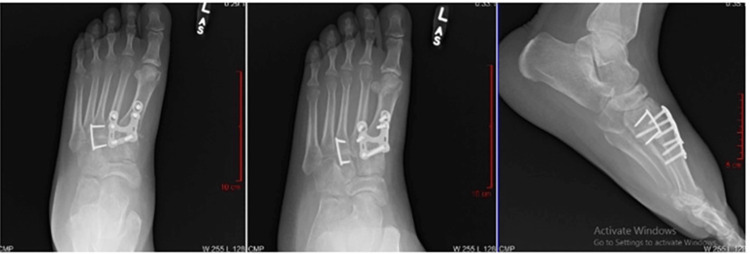
Example of mock patient scenario (Case 5) provided to surgeons; Post-operative radiographs demonstrating midfoot arthrodesis.

The scenarios included pre-operative diagnostic imaging and reports, intra-operative imaging, and post-operative radiographs (Figures 1-4).

For each patient scenario, surgeons were asked which CPT codes would be applied and if any CPT modifiers would be used. If multiple CPT codes were used, surgeons were asked to list the codes in the same order as would be theoretically listed on their operative notes. Current Procedural Terminology codes and modifiers were not part of a prepopulated response set, and surgeons could respond freely. There was no limit to how many codes could be applied, respondents were allowed to use any sources of information to complete the survey, and there was no time limit.

Total work-relative value units (RVUs) and the generated reimbursement values were calculated using the 2024 Centers for Medicare & Medicaid Services (CMS) conversion factor ($32.74 per RVU), with the primary procedure reimbursed at 100% and additional procedures reimbursed at 50%. Total RVUs and generated reimbursement were calculated when both the primary procedure was listed in the same order the surgeons listed (uncorrected), as well as when the primary procedure was adjusted to be the highest RVU procedure (corrected). The differences in reimbursement between corrected and uncorrected RVUs were calculated among all 12 cases for each respondent.

Descriptive statistics were calculated for the number of CPT codes and modifiers used for each patient-case scenario. Coefficients of variation were used to quantify variability in the number of CPT codes listed in each case. All statistics were performed using Microsoft Excel (version 2501 for Windows, Microsoft Corporation, Redmond, WA).

## Results

Five fellowship-trained foot-and-ankle surgeons with median years of practice of 11 years (range, three to 29 years) completed the survey, for a response rate of 100%. Only four of the 12 patient cases (33.3%) had at least four of the five respondents agreeing on the primary CPT code, whereas only one case (8.3%, Case 4) had 100% agreement. Case 1 (second metatarsal head overload), case 5 (Lisfranc injury), Case 6 (Maisonneuve injury), Case 8 (Charcot midfoot reconstruction), and Case 9 (midfoot fracture-dislocations) had the greatest variation in number of CPT codes used (Table 2), with all having coefficients of variation greater than 40%.

**Table 2:** Total RVUs and corrected reimbursement among survey respondents RVU: work-relative value units

Respondent	Total RVUs in 12 cases	Reimbursement ($)
1	282.12	6685.18
2	484.93	9833.95
3	342.81	7605.67
4	498.18	10313.10
5	371.62	8078.60

The median number of modifiers listed for each case ranged from 0 (Cases 1, 4, 6, and 12) to 5 (Cases 8 and 9). Modifier 59 was the most used, followed by Modifiers 51 and 22. Modifier 59 described a distinct procedural service being performed on the same day or during the same procedure. Modifier 51 described multiple procedures performed at the same time and was applied to the lower RVU procedures. Modifier 22 described increased procedural services when the work required to provide a service was greater than typically required [5,6].

Similar to CPT code differences, only five of the 12 cases (41.7%) had at least four of the five respondents in agreement regarding modifier usage, with only one case (8.3%, Case 4) having 100% agreement. The total RVU and corrected reimbursement values for each respondent are displayed in Table 3. The RVU and reimbursement difference between the respondents with the highest (Respondent 4) and lowest (Respondent 1) listed RVUs was 216.06 and $3,627.92, respectively.

**Table 3:** RVU difference between corrected and uncorrected primary procedures RVU: work-relative value units

Respondent	Uncorrected reimbursement ($)	Difference vs. Corrected primary code ($)
1	6618.39	-66.79
2	9691.53	-142.42
3	7567.69	-37.98
4	10313.10	0
5	7995.44	-83.16

In total, there were eight of 60 instances (13.3%) where the first-listed CPT did not have the highest RVU of listed CPT codes. The uncorrected reimbursement values and differences versus the corrected primary procedure are listed in Table 4.

**Table 4:** RVU difference between corrected and uncorrected primary procedures RVU: work-relative value units

Respondent	Uncorrected reimbursement ($)	Difference vs. Corrected primary code ($)
1	6618.39	-66.79
2	9691.53	-142.42
3	7567.69	-37.98
4	10313.10	0
5	7995.44	-83.16

## Discussion

We have demonstrated that procedural coding can vary widely among common foot-and-ankle cases; a detailed understanding of it can improve efficiency and ultimately lead to higher compensation. It is necessary to be able to properly document and charge for services provided, and the implementation of appropriate codes is necessary to prevent financial loss, as evidenced by the potential compensation losses in our study. Accuracy of coding is also needed to avoid over- or underbilling or fraud, which can lead to delays in payment while errors are corrected, as well as possible penalties by the Department of Health and Human Services [2,9]. Even if surgeons use professional coders, the surgeons must be knowledgeable about coding and billing since they will be legally and ethically responsible for any billing performed under their names [9].

This investigation can be treated as a small-scale version of the variations seen in an orthopedic practice. With only 12 patient cases of common foot-and-ankle cases and procedures, we demonstrated a potential RVU difference of 216.06 and a possible reimbursement difference of $3,627.92. Only one case had uniform (100%) agreement on the primary CPT code used, while only four of the 12 cases had 80% agreement. When extrapolating these differences over months and years, the variations can cause substantial financial implications for an orthopedic practice. Compared with joint arthroplasty surgeons, who often only require a few codes to describe their entire clinical practice, foot-and-ankle surgeons must involve various bone, joint, tendon, ligament, and nerve codes, similar to their hand-surgery counterparts [9]. Thus, coding education may be more crucial for these providers, especially in a large multi-subspecialty orthopedic practice. The AOFAS offers in-person coding and billing courses as well as other online resources for practicing surgeons [13].

In our survey, the cases with the most variability included a second metatarsal head overload undergoing Weil osteotomy, Lisfranc injury undergoing arthrodeses, Maisonneuve injury fixation, Charcot midfoot reconstruction, and midfoot fracture-dislocations undergoing multiple arthrodeses. These differences may be in part due to variable surgeon techniques in these procedures, misinterpretation of procedures performed given the pre- and postoperative imaging, or inaccurate coding. Most of these cases involved the midfoot, a region with many joints and the potential for large variability in procedures performed.

Previous studies have demonstrated variations in coding practice among orthopedic surgeons, though not in foot-and-ankle surgery. Iobst et al. demonstrated substantial coding variation in a survey consisting of 10 common patient cases completed by 34 experienced pediatric limb reconstruction surgeons in the United States [10]. They found only three of the 10 cases had an agreement greater than 75% for any single code, and only two cases had greater than 50% agreement on any combination of two codes. In each case, the number of unique codes submitted ranged from five to 20.

Similar results have been found in the hand literature. In their study of 421 hand surgeons in the American Society for Surgery of the Hand, Coyle et al. demonstrated that surgeons in a collections-based model coded for significantly higher RVUs on average compared with surgeons in a fixed salary and RVU-based model in one of four commonly encountered hypothetical hand surgery cases. The authors emphasized the need for improved communication and education among surgeons regarding appropriate coding practices and more easily available coding reference material [8]. Lifchez et al. also found significant differences in coding in their survey of six hypothetical hand surgery cases among residents, attending surgeons, and professional coders [9].

Increased emphasis has been placed on coding education during orthopedic residency and fellowship training in recent years so future surgeons can be prepared when entering practice [6,9,14]. Coding falls within the Accreditation Council for Graduate Medical Education (ACGME)’s core competencies of Professionalism and Systems-Based Practice [9], so it should be considered an essential part of medical training. In their recent survey of orthopedic residents, Stautberg et al. reported that 97% of residents desired coding to be taught, and 80% reported having “no confidence” or “some confidence” in coding operative cases [15]. Additionally, 42% of respondents reported having no lecture or grand rounds discussing CPT codes for billing operative cases.

Murphy et al. reported that 0% of residents who had completed a foot-and-ankle rotation were “very comfortable” logging CPT codes, with 43% reporting they were “uncomfortable” on a five-point Likert scale. All three attending surgeons in the study reported being “somewhat comfortable” with foot-and-ankle CPT codes [11]. In their mock coding survey, Greenky et al. reported a significantly higher coding accuracy among practicing surgeons compared with senior and junior residents. Any coding education was associated with a significantly improved overall score for residents included in the study [2]. In a study of pediatric orthopedic fellows receiving hypothetical case scenarios, large variability in coding was demonstrated, with percentage differences across all scenarios ranging from 29% to 100% among 46 fellows responding [16].

Limitations of the current study included a low respondent number and all surgeons being a part of the same academic-private practice orthopedic group, which might have biased the responses. Future studies should aim to include surgeons in different practice settings, compensation structures, and geographic locations to see the differences these factors may contribute to coding practices. Additional investigations should also include non-fellowship-trained orthopedic surgeons as well as resident surgeons. A standardized reimbursement value was generated for this study that may substantially differ from compensation models in other locations and practices. Thus, the values generated in our study may not have high external generalizability; however, attempting to incorporate all surgeon compensation models in any coding study is not practical. The actual reimbursement received for the included real-life patient cases was also not reported, but this is similarly unlikely to be generalizable to all practicing surgeons. Case descriptions may not have been detailed to determine what procedures were specifically performed, but we believe the pre- and post-operative patient imaging provided, combined with the experience of our responding surgeons, allowed for enough direction for them to generate appropriate coding.

## Conclusions

This study demonstrated the large variability that exists between fellowship-trained foot-and-ankle orthopedic surgeons when coding common procedures, proving our hypothesis. Current Procedural Terminology codes, modifier usage, and calculated reimbursement values all varied among surgeons in our mock survey. Surgeons should be particularly aware that procedures involving the midfoot can be sources of large coding variations and should pay special attention when coding these cases. Increasing competency with coding and billing, starting in residency training and continuing into practice, should continue to be emphasized in all medical specialties.
